# A Homemade Autosampler/Injector Commutator for Flow Injection Analysis

**DOI:** 10.1155/JAMMC/2006/42987

**Published:** 2006

**Authors:** Eduardo Costa de Figueiredo, Leandro Ruela de Souza, Cristiana Schmidt de Magalhães, Célio Wisniewski, Pedro Orival Luccas

**Affiliations:** 1 Departamento de Ciências Exatas Universidade Federal de Alfenas (UNIFAL-MG) 714 Rua Gabriel Monteiro da Silva Alfenas MG CEP 37130-000 Brazil

## Abstract

An autosampler/injector commutator for flow injection analysis (FIA) was constructed with electronic components of used equipments. The apparatus is controlled by commercially available multifunctional interface (PCL711B) connected to a personal computer, and the software was written in Visual Basic language. The system was applied to water analysis and it presented satisfactory results. The low cost and simplicity are the principal characteristics of the autosampler/injector commutator.


Hindawi Publishing Corporation
Journal of Automated Methods and Management in Chemistry
Volume 2006, Article ID 42987, Pages 1–4
DOI 10.1155/JAMMC/2006/42987

### A Homemade Autosampler/Injector Commutator for Flow Injection Analysis

**Eduardo Costa de Figueiredo, Leandro Ruela de Souza, Cristiana Schmidt de Magalhães, Célio Wisniewski, and Pedro Orival Luccas**

*Departamento de Ciências Exatas, Universidade Federal de Alfenas (UNIFAL-MG), 714 Rua Gabriel Monteiro da Silva, CEP 37130-000, Alfenas, MG, Brazil*

Received 21 November 2005; Revised 30 January 2006; Accepted 3 April 2006

An autosampler/injector commutator for flow injection analysis (FIA) was constructed with electronic components of used equipments. The apparatus is controlled by commercially available multifunctional interface (PCL711B) connected to a personal computer, and the software was written in Visual Basic language. The system was applied to water analysis and it presented satisfactory results. The low cost and simplicity are the principal characteristics of the autosampler/injector commutator.

Copyright © 2006 Eduardo Costa de Figueiredo et al. This is an open access article distributed under the Creative Commons Attribution License, which permits unrestricted use, distribution, and reproduction in any medium, provided the original work is properly cited.

### 1. INTRODUCTION

In analytical chemistry, as well as in all the other areas of sciences, new tools that made the analyst's work easier and decreased the operation time have been produced [1]. In this context, great emphasis has been attributed to the flow injection analysis (FIA) systems due to their vast uses in several techniques such as electrochemistry [2], atomic absorption spectrometry [3], atomic emission spectrometry [4], chromatography [5], and spectrophotometry [6], among others.

FIA system is based on the sample introduction into a carrier stream, which is transported until a detector [7]. During the sample course, reagents can be added either as chromogenic reagents or as another function such as interferents elimination, pH adjust, and ionic force, among others. Several advantages of the FIA system can be related, such as the raise in analytical frequency, low reagents and solutions consumption, and mainly, its versatility [8]. In this way, the creativity of many researchers has been wakening up resulting in several homemade devices. As an example, Bergamin et al. [9] improved the analytical performance of a flow system using an injector commutator (IC) as a sample introduction device. In 1980, the same researchers mechanized an IC [10] using two solenoids to switch the sampling and injection positions.

Nowadays, the computer is an ordinary apparatus in many laboratories, interfacing almost all the analytical instruments [11]. The benefits of the computer use are unquestionable, mainly by the partnership of computers and flow systems. According to Pasquini and Faria [12], there are four possible points of automation in the FIA systems: (1) sample injection, (2) sample presentation for analysis, (3) detector control, and (4) data acquisition interface. The authors established their ideas showing a system where the sample introduction was done through a solenoid valve, and they used a commercially available autosampler for sample presentation. CI 8155 I/O and a CI 74LS373 were used as interface and buffer, respectively, and the programming language employed was Assembler.

Another interesting example of flow automation is the work of Prop et al. [13], where the system proposed could work for several hours without analyst's intervention. A commercially autosampler, equipped with solenoid valve, was controlled by computer program (written in Basic language) using a parallel interface.

Flow system automation procedures are well accepted in analytical chemistry. This is due the potentialities attributed to the FIA, as well as the demand of laboratories to high quantities of results. Thus, the present work aim is to build an autosampler/injector commutator automated for FIA systems using simple devices commonly found in electronic equipments. The software was written in Visual Basic 6.0 language and a commercially multifunctional interface (PCL711, Advantech) was used to control the system.

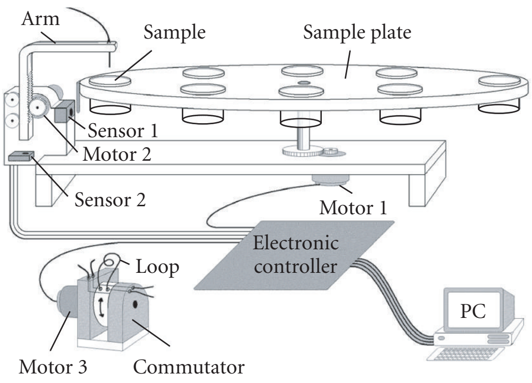

Figure 1: General scheme of the autosampler and injector commutator.

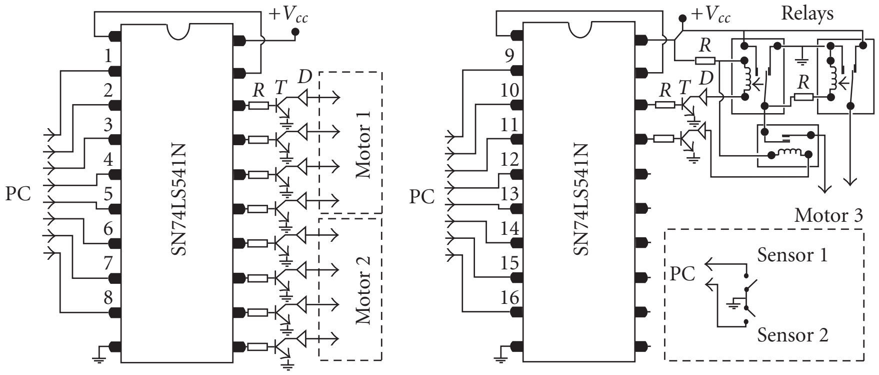

Figure 2: General scheme of electronic controller. The relays are commercial devices: 12 V, 10 A : 250 V; $R = 300\Omega$, $T$ = TIP122, and $D$ = 1N4348.

### 2. MATERIALS AND METHODS

The salvage parts from scraps instruments used in this work are listed below:

(i) 2 stepping motors (1 and 2-HSI 31616-31) from old atomic absorption equipment;
(ii) 1 DC motor (3-commercial DC motor-12 V, 1 A) from automobile glass;
(iii) 2 sensors (electrical switches) from mouse of computer;
(iv) 1 sample injector arm built with acrylic of an old electrophoresis cube;
(v) 1 sample plate with 8 samples compartment built with acrylic of an old electrophoresis cube.

The autosampler (Figure 1) allows the analysis of 8 samples sequentially substituted during the process. The system is controlled by a microcomputer through a 12- bit analogical-digital interface (PCL 711B-Advantech) endowed with 12 bidirectional digital channels that get/put information from/ to an electronic controller, it is connected to the autosampler (motors and sensors).

As can be seen in Figure 1, the components of the autosampler are 2 stepping motors (1 and 2-HSI 31616-31), 1 DC motor (3-commercial DC motor-12 V, 1 A), 2 sensors (electrical switches), 1 sample injector arm, 1 sample plate with 8 samples compartment, and an electronic controller, as shown in Figure 2. Sample 1 is adjusted in the first position by sensor 1 during a 360° sample plate rotation and the arm starting position (lifted up) is gotten from down position (sensor 2 response) after an arm vertical shift. Between the plate axis and stepping motor 1, there is mechanic reduction torque (1:1000) to increase the rotation precision of sample plate (stepping/radians).

The injector commutator has two positions: sampling (A) and injection (B) of sample loop. A DC motor was used to commute this device due to the high torque and speed of response. The inversion of rotation direction can be obtained by reversal electric polarity.

The electronic controller is responsible for carrying out the commands from microcomputer. This device has 2 integrated components SN74LS541N (or similar), which provide isolation and protection to PCL 711-B and adapt the power to TIP 122 transistors. These components control the

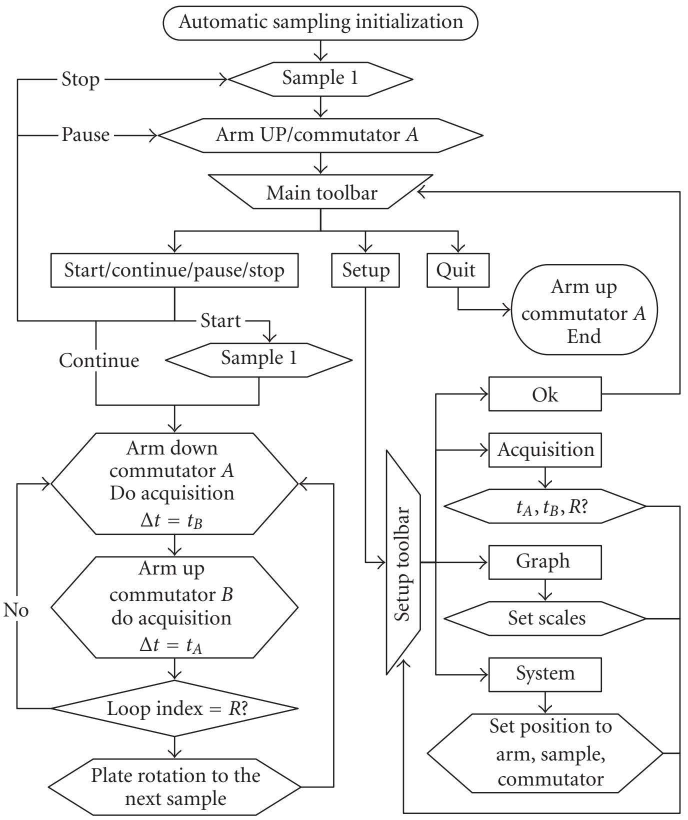

Figure 3: Flowchart for automatic sampling software: $\Delta t$ = time interval, $t_A$ = time to inject sample in the system, $t_B$ = time to fill sample loop, and $R$ = repetitions number.

stepping motors directly and switch relays to control the DC motor.

The rotation direction and speed of motors are attributed to software, as well as the signal acquisition from others instruments just as spectrophotometers and voltmeters. *Visual Basic for Windows 6.0* and *Microsoft Windows 98* were used as a compiler and an operational system, respectively. The flowchart of the program is shown in Figure 3. The first step is the initialization of variables and the automatic sampling system positioning. The commutator is set up to "*A*" position, the arm at "UP" (sensor 2 event) position, and the plate on first sample (sensor 1 event). After the setup of graph and acquisition variables, the user can carry out the acquisition. The acquisition setup has two main variables: time to fill ($t_A$) and to inject ($t_B$) of the sample loop into system, besides the number of repetitions $R$ (where time is equal to $t_A + t_B$) between sample exchanges and the number of samples can be attributed to. All parts of autosampler are made from scrap instruments, therefore it has low costs.

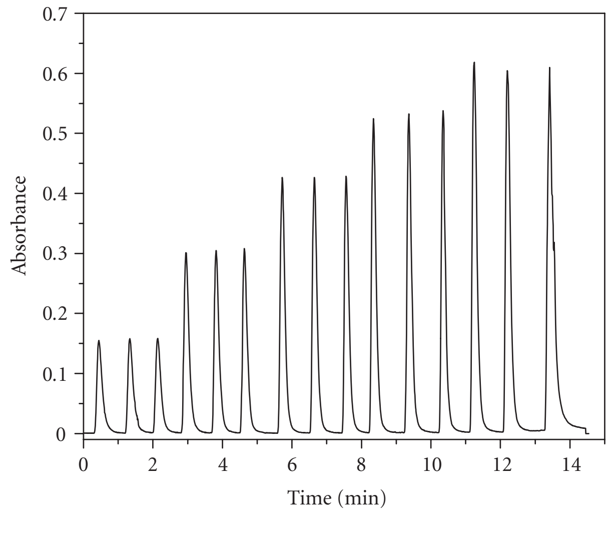

Figure 4: Analytical signals (in triplicate) of 5, 10, 15, 20, 25 mg $L^{-1}PO_4^{3-}$ standard solutions.

### 3. APPLICATION

The autosampler and injector commutator proposed in the present work was tested in determination of phosphate in water [14]. Figure 4 shows the signal registerations, in triplicate, for standard solutions. The measurements were done in peak height. The linear regression equation for the analytical curve was $A = 0.0229C + 0.0581$ (A= absorbance in peak height and $C = PO_4^{-3}$ concentration in mg$L^{-1}$) and the linear correlation coefficient obtained was $R = 0.9937$. The good precision of the measurements with relative standard deviation was always lower than 1.4%.

### 4. CONCLUSION

The autosampler and injector commutator presented satisfactory results. Its main characteristics are low cost for building and implementation, robustness, and easy operation. It is important to point out that some components were obtained from broken electronic equipments (scraps). The approximate cost of the components used was of 50 dollars.

### ACKNOWLEDGMENTS

Financial support from Coordenção de Aperfeiiçoamento de Pessoal de Nível Superior (CAPES), Financiadora de Estudos e Projetos (FINEP), and also from the CNPq Scholarship granted to the first author is gratefully acknowledged.

### REFERENCES

[1] V. Cerdá, J. M. Estela, R. Forteza, et al., "Flow techniques in water analysis," *Talanta*, vol. 50, no. 4, pp. 695–705, 1999.

[2] R. I. L. Catarino, M. B. Q. Garcia, J. L. F. C. Lima, E. Barrado, and M. Vega, "Relocation of a tubular voltammetric detector for standard addition in FIA," *Electroanalysis*, vol. 14, no. 11, pp. 741–746, 2002.

[3] C. R. T. Tarley and M. A. Z. Arruda, "A sensitive method for cadmium determination using an on-line polyurethane foam preconcentration system and thermospray flame furnace atomic absorption spectrometry," *Analytical Sciences*, vol. 20, no. 6, pp. 961–966, 2004.

[4] C. Ortega, M. R. Gomez, R. A. Olsina, M. F. Silva, and L. D. Martinez, "On-line cloud point preconcentration and determination of gadolinium in urine using flow injection inductively coupled plasma optical emission spectrometry," *Journal of Analytical Atomic Spectrometry*, vol. 17, no. 5, pp. 530–533, 2002.

[5] A. Tolokán, I. Klebovich, K. Balogh-Nemes, and G. Horvai, "Automated determination of levodopa and carbidopa in plasma by high-performance liquid chromatography-electrochemical detection using an on-line flow injection analysis sample pretreatment unit," *Journal of Chromatography B: Biomedical Sciences and Applications*, vol. 698, no. 1-2, pp. 201–207, 1997.

[6] J. L. Rodrigues, C. S. de Magalhães, and P. O. Luccas, "Flow injection spectrophotometric determination of Al in hemodialysis solutions," *Journal of Pharmaceutical and Biomedical Analysis*, vol. 36, no. 5, pp. 1119–1123, 2005.

[7] J. Ruzicka and E. H. Hansen, *Flow Injection Analysis*, John Wiley & Sons, New York, NY, USA, 1981.

[8] A. F. Danet, M. Cheregi, J. M. Calatayud, J. V. G. Mateo, and H. Y. Aboul Enein, "Flow injection methods of analysis for waters. I. Inorganic species," *Critical Reviews in Analytical Chemistry*, vol. 31, no. 3, pp. 191–222, 2001.

[9] H. Bergamin, E. A. G. Zagatto, F. J. Krug, and B. F. Reis, "Merging zones in flow injection analysis: part 1. Double proportional injector and reagent consumption," *Analytica Chimica Acta*, vol. 101, no. 1, pp. 17–23, 1978.

[10] H. Bergamin, B. F. Reis, A. O. Jacintho, and E. A. G. Zagatto, "Ion exchange in flow injection analysis. Determination of ammonium ions at the $\mu$g $1^{-1}$ level in natural waters with pulsed Nessler reagent," *Analytica Chimica Acta*, vol. 117, no. 1, pp. 81–89, 1980.

[11] D. J. Malcome-Lawes, *Microcomputer and Laboratory Instrumentation*, Plenum Press, New York, NY, USA, 2nd edition, 1998.

[12] C. Pasquini and L. C. De Faria, "Operator-free flow injection analyser," *Journal of Automatic Chemistry*, vol. 13, no. 4, pp. 143–146, 1991.

[13] L. T. M. Prop, P. C. Thijssen, and L. G. G. van Dongen, "A software package for computer-controlled flow-injection analysis," *Talanta*, vol. 32, no. 3, pp. 230–234, 1985.

[14] J. M. Estela and V. Cerdá, "Flow analysis techniques for phosphorus: an overview," *Talanta*, vol. 66, no. 2, pp. 307–331, 2005.

